# Supplementary material for: Dynamics of miRNA driven feed-forward loop depends upon miRNA action mechanisms
Source: BMC Genomics. 2014 Dec 19;15(Suppl 12):S9. doi: 10.1186/1471-2164-15-S12-S9 (PMC4303955; doi:10.1186/1471-2164-15-S12-S9)
Supplement: Additional file 1 — contains details of mathematical analysis of the coupled ODE. [file 1471-2164-15-S12-S9-S1.PDF]

1 Mathematical analysis of equations governed the miRNA driven FFL behaviour

Rigorous mathematical statements may form a solid basis for results and conclusions in the area of application. They might be called as mathematical debris, however, without it most of biological interpretations and conclusions would be just words to be taken for granted. For this reason we describe main steps of transformations in this section.

For reader's convenience we re-write the basic equations of the problem, namely, the five coupled differential equations proposed in the paper by Osella et al, (2011) for an incoherent feed-forward loop (FFL).

$$\begin{cases} \dfrac{dw}{dt} = k_w - g_w w, & (mRNA\,of\,TF) \\ \dfrac{dq}{dt} = k_q w - g_q q, & (TF) \\ \dfrac{ds}{dt} = k_s(q) - g_s s, & (miRNA) \\ \dfrac{dr}{dt} = k_r(q) - g_r r, & (mRNA) \\ \dfrac{dp}{dt} = k_p(s) r - g_p p. & (target\,protein) \end{cases} . \tag{1}$$

Here k_w and k_q are rates of TF mRNA and TF synthesis, $k_s(q)$ and $k_r(q)$ are rates of transcription of the regulated gene, $k_p(s)$ is the rate of target protein synthesis; g_w, g_q,g_s,g_s and g_p represent the degradation rates of the corresponding species.

1.1 Exact solutions under Stop model

Let the time variable be in the interval $t \in [0, 1]$, then for each of unknowns $x \in (w, q, s, r, p)$ we prescribe the boundary conditions in the form: $x|_{t=0} = A; \quad x|_{t=1} = B$, with constants A, B given for every x.

Both first and second equations of system (1) can be easily integrated in closed form, that leads to the following expression for the number of TF mRNA molecules:

$$w = w_0 \exp(-g_w t) + \frac{k_w}{g_w} \left(1 - \exp(-g_w t)\right), \tag{2}$$

as well as for the number of TF molecules:

$$q = a_q \exp(-g_w t) + b_q \exp(-g_q t) + c_q, \tag{3}$$

where the following quantities were introduced:

$$a_q = \frac{k_q}{g_q - g_w}\left(w_0 - \frac{k_w}{g_w}\right); \quad b_q = q_0 - c_q - a_q \quad c_q = \frac{k_q k_w}{g_q g_w}. \tag{4}$$

In order to solve the third equation in (1) for the number q of miRNA molecules one has to evaluate an integral:

$$\int \frac{dz}{h_s^2 + (a_q z^{-g_w/g_s} + b_q z^{-g_q/g_s} + c_q)^2}, \tag{5}$$

which can be found in closed form only for several integer powers, denoted in (5) as: g_w/g_s, g_q/g_s, while should be numerically calculated otherwise. Fortunately, some integer values correspond to problems of biological interest.

As an example, we firstly consider the values $g_w = 2g_q, g_s = g_q$, which mean that the TF mRNA degrades *two times* faster, than both the TF and the miRNA. In this case (5) has the form:

$$\int \frac{dz}{h_s^2 + (a_q z^{-2} + b_q z^{-1} + c_q)^2} = \int \frac{z^4 dz}{a_s z^4 + b_s z^3 + c_s z^2 + d_s z + e_s}, \tag{6}$$

where:

$$a_s = h_s^2 + c_q^2; b_s = 2b_q c_q; c_s = b_q^2 + 2a_q c_q; d_s = 2a_q b_q; e_s = a_q^2. \tag{7}$$

Upon some algebra the last integral can be decomposed and calculated in closed form. Details of further computations for this and other relationships between the degradation rates can be found below. Therefore the solutions to the coupled equations (1) can be found consequently and in closed form, however the final expressions are cumbersome. Moreover, the uniqueness of the solutions was proven, too, see below.

Both the solutions to the non-linear ODE and their uniqueness allow us to make a rigorous statement: we found the unique exact solution to (1) for any combination of initial conditions and values of parameters, and over the whole time interval $[0, T]$ given.

1.2 The integral calculation

Assume firstly that $g_q = g_s$, and the miRNA degradation is comparatively slow.

We have to calculate the following integral:

$$\int \frac{dz}{h_s^2 + (a_q z^{-2} + b_q z^{-1} + c_q)^2} = \int \frac{z^4 dz}{a_s z^4 + b_s z^3 + c_s z^2 + d_s z + e_s}, \tag{8}$$

where:

$$a_s = h_s^2 + c_q^2; b_s = 2b_q c_q; c_s = b_q^2 + 2a_q c_q; d_s = 2a_q b_q; e_s = a_q^2. \tag{9}$$

Assume that $z_{1,2,3,4}$ are the roots of the 4th order polynomial in the denominator and decompose the integral (8) into the following form:

$$\begin{aligned} \int \frac{z^4 dz}{a_s z^4 + b_s z^3 + c_s z^2 + d_s z + e_s} &= \int \frac{dz}{a_s} - \\ -\frac{1}{a_s^2}\left(\int \frac{\alpha_1 + \alpha_2 i}{z - z_1} dz + \int \frac{\beta_1 + \beta_2 i}{z - z_2} dz + \int \frac{\gamma_1 + \gamma_2 i}{z - z_3} dz + \int \frac{\delta_1 + \delta_2 i}{z - z_4} dz \right). \end{aligned} \tag{10}$$

Writing down the complex roots z_i as:

$$z_1 = \xi_1 + \xi_2 i, z_2 = \xi_3 + \xi_4 i, z_3 = \xi_1 - \xi_2 i, z_4 = \xi_3 - \xi_4 i, \tag{11}$$

where:

$$\begin{aligned} \xi_1 &= \frac{-b_q c_q - c_q\sqrt{r_D}\cos\frac{\phi}{2} - h_s\sqrt{r_D}\sin\frac{\phi}{2}}{2c_q^2 + 2h_s^2}; \quad \xi_2 = \frac{h_s b_q + h_s\sqrt{r_D}\cos\frac{\phi}{2} - c_q\sqrt{r_D}\sin\frac{\phi}{2}}{2c_q^2 + 2h_s^2}; \\ \xi_3 &= \frac{-b_q c_q + c_q\sqrt{r_D}\cos\frac{\phi}{2} + h_s\sqrt{r_D}\sin\frac{\phi}{2}}{2c_q^2 + 2h_s^2}; \quad \xi_4 = \frac{h_s b_q - h_s\sqrt{r_D}\cos\frac{\phi}{2} + c_q\sqrt{r_D}\sin\frac{\phi}{2}}{2c_q^2 + 2h_s^2}, \\ r_D &= \sqrt{(b_q^2 - 4a_q c_q)^2 + 16a_q^2 h_s^2}; \ \tan\phi = -4a_q h_s/(b_q^2 - 4a_q c_q), \end{aligned} \tag{12}$$

and the terms of complex expressions in numerators in (10) as:

$$\begin{aligned}
\alpha_1 &= u_1 - \frac{(u_3 - \xi_3 u_1 + \xi_4 u_2)(\xi_1 - \xi_3) + (u_4 - \xi_4 u_1 - \xi_3 u_2)(\xi_2 - \xi_4)}{(\xi_1 - \xi_3)^2 + (\xi_2 - \xi_4)^2} \\
\alpha_2 &= u_2 - \frac{(u_4 - \xi_4 u_1 - \xi_3 u_2)(\xi_1 - \xi_3) - (u_3 - \xi_3 u_1 + \xi_4 u_2)(\xi_2 - \xi_4)}{(\xi_1 - \xi_3)^2 + (\xi_2 - \xi_4)^2} \\
\beta_1 &= \frac{(u_3 - \xi_3 u_1 + \xi_4 u_2)(\xi_1 - \xi_3) + (u_4 - \xi_4 u_1 - \xi_3 u_2)(\xi_2 - \xi_4)}{(\xi_1 - \xi_3)^2 + (\xi_2 - \xi_4)^2} \\
\beta_2 &= \frac{(u_4 - \xi_4 u_1 - \xi_3 u_2)(\xi_1 - \xi_3) - (u_3 - \xi_3 u_1 + \xi_4 u_2)(\xi_2 - \xi_4)}{(\xi_1 - \xi_3)^2 + (\xi_2 - \xi_4)^2} \\
\gamma_1 &= \alpha_1; \gamma_2 = -\alpha_2; \delta_1 = \beta_1; \delta_2 = -\beta_2,
\end{aligned} \tag{13}$$

where:

$$u_1 = b_q c_q;\ u_2 = b_q(c_q^2 - h_s^2)/(2h_s);\ u_3 = -a_q c_q;\ u_4 = a_q(h_s^2 - c_q^2)/(2h_s), \tag{14}$$

we are able to calculate the integral in closed form. For example, the first term in (10) yields:

$$\int \frac{\alpha_1 + \alpha_2 i}{z - (\xi_1 + \xi_2 i)} dz = (\alpha_1 + \alpha_2 i) \log \sqrt{(z - \xi_1)^2 + \xi_2^2} + (\alpha_1 i - \alpha_2) \arctan\left(\frac{z - \xi_1}{\xi_2}\right)$$

The imaginary part in this and similar expressions will be diminished, and eventually *the exact solution* to the equation for the number s of the miRNA molecules in time t with an initial value s_0 has the following form:

$$\begin{aligned}
s = {} & s_0 \exp(-g_s t) + \frac{k_s c_q^2}{g_s(c_q^2 + h_s^2)}(1 - \exp(-g_s t)) + \frac{\exp(-g_s t) k_s h_s^2}{g_s(c_q^2 + h_s^2)^2} \cdot \\
& \cdot \left[\alpha_1 \log\left(\frac{(\exp(g_s t) - \xi_1)^2 + \xi_2^2}{(1 - \xi_1)^2 + \xi_2^2}\right) - 2\alpha_2 \arctan\left(\frac{\xi_2(\exp(g_s t) - 1)}{\xi_1^2 + \xi_2^2 - \xi_1(1 + \exp(g_s t)) + \exp(g_s t)}\right)\right. \\
& \left. + \beta_1 \log\left(\frac{(\exp(g_s t) - \xi_3)^2 + \xi_4^2}{(1 - \xi_3)^2 + \xi_4^2}\right) - 2\beta_2 \arctan\left(\frac{\xi_4(\exp(g_s t) - 1)}{\xi_3^2 + \xi_4^2 - \xi_3(1 + \exp(g_s t)) + \exp(g_s t)}\right)\right].
\end{aligned} \tag{15}$$

When the degradation coefficients are defined as follows: $g_w = g_s = 2g_q$, we can find the *exact solution* for the *fast miRNA degradation* in a similar way. The solution will have the following form:

$$\begin{aligned}
s = {} & s_0 \exp(-g_s t) + \frac{k_s c_q^2}{g_s(h_s^2 + c_q^2)} (1 - \exp(-g_s t)) + \frac{4 b_q c_q k_s h_s^2}{g_s(c_q^2 + h_s^2)^2} (\exp(-g_s t/2) - \exp(-g_s t)) + \\
& + \frac{2 k_s h_s^2 \exp(-g_s t)}{g_s(c_q^2 + h_s^2)^3} \left[\hat{\alpha}_1 \log\left(\frac{(\exp(g_s t/2) - \xi_1)^2 + \xi_2^2}{(1 - \xi_1)^2 + \xi_2^2}\right) + \hat{\beta}_1 \log\left(\frac{(\exp(g_s t/2) - \xi_3)^2 + \xi_4^2}{(1 - \xi_3)^2 + \xi_4^2}\right) + \right. \\
& - 2\hat{\alpha}_2 \arctan\left(\frac{\xi_2(\exp(g_s t/2) - 1)}{\xi_1^2 + \xi_2^2 - \xi_1(1 + \exp(g_s t/2)) + \exp(g_s t/2)}\right) \\
& \left. - 2\hat{\beta}_2 \arctan\left(\frac{\xi_4(\exp(g_s t/2) - 1)}{\xi_3^2 + \xi_4^2 - \xi_3(1 + \exp(g_s t/2)) + \exp(g_s t/2)}\right)\right].
\end{aligned} \tag{16}$$

The values $\hat{\alpha}_{1,2}, \hat{\beta}_{1,2}$ can be found by substitution of quantities $\hat{u}_{1,2,3,4}$ in (13) written in the form:

$$\begin{aligned} &\hat{u}_1 = a_q c_q (c_q^2 + h_s^2) - \frac{b_q^2}{2}(3c_q^2 - h_s^2); \hat{u}_2 = \frac{a_q(c_q^2 - h_s^2)}{2h_s}(c_q^2 + h_s^2) - \frac{c_q b_q^2}{2h_s}(c_q^2 - 3h_s^2); \\ &\hat{u}_3 = \frac{a_q b_q}{2}(3c_q^2 - h_s^2); \hat{u}_4 = \frac{a_q b_q c_q}{2h_s}(c_q^2 - 3h_s^2). \end{aligned} \tag{17}$$

instead of $u_{1,2,3,4}$.

Obviously, the stationary (steady state) solutions will be identical in both cases, and they can be written as:

$$s_{stat} = \frac{k_s}{g_s} - \frac{k_s h_s^2}{g_s(c_q^2 + h_s^2)} = \frac{k_s c_q^2}{g_s(c_q^2 + h_s^2)} = \frac{k_s k_q^2 k_w^2}{g_s(k_q^2 k_w^2 + h_s^2 g_q^2 g_w^2)}. \tag{18}$$

We can calculate an analytical solution to the problem with *very fast* miRNA degradation, when $g_w = g_s = 3g_q$, in a similar way, however, it contains coefficients, which are too lengthy to write them here.

The exact solution to the fourth equation $dr/dt = k_r(q) - g_r r$ for target mRNA can be found in the same way, as the solution to the third equation for miRNA.

We can calculate it for both the slow and the fast target mRNA degradation rate. Upon several transformations we have the following *exact solution* for slow degradation of mRNA:

$$\begin{aligned} r = {} & r_0 \exp(-g_r t) + \frac{k_r h_r^2}{g_r(c_q^2 + h_r^2)}(1 - \exp(-g_r t)) - \frac{\exp(-g_r t) k_r h_r^2}{g_r(c_q^2 + h_r^2)^2} \cdot \\ & \cdot \left[\tilde{\alpha}_1 \log\left(\frac{(\exp(g_r t) - \tilde{\xi}_1)^2 + \tilde{\xi}_2^2}{(1 - \tilde{\xi}_1)^2 + \tilde{\xi}_2^2}\right) - 2\tilde{\alpha}_2 \arctan\left(\frac{\tilde{\xi}_2(\exp(g_r t) - 1)}{\tilde{\xi}_1^2 + \tilde{\xi}_2^2 - \tilde{\xi}_1(1 + \exp(g_r t)) + \exp(g_r t)}\right)\right. \\ & \left. + \tilde{\beta}_1 \log\left(\frac{(\exp(g_r t) - \tilde{\xi}_3)^2 + \tilde{\xi}_4^2}{(1 - \tilde{\xi}_3)^2 + \tilde{\xi}_4^2}\right) - 2\tilde{\beta}_2 \arctan\left(\frac{\tilde{\xi}_4(\exp(g_r t) - 1)}{\tilde{\xi}_3^2 + \tilde{\xi}_4^2 - \tilde{\xi}_3(1 + \exp(g_r t)) + \exp(g_r t)}\right)\right] \end{aligned} \tag{19}$$

Similarly, for *fast degradation* rate we have another exact solution to r in the problem:

$$\begin{aligned} r = {} & r_0 \exp(-g_r t) + \frac{k_r h_r^2}{g_r(c_q^2 + h_r^2)}(1 - \exp(-g_r t)) - \frac{4k_r h_r^2 b_q c_q}{g_r(h_r^2 + c_q^2)^2}(\exp(-g_q t) - \exp(-g_r t)) - \\ & - \frac{2k_r h_r^2 \exp(-g_r t)}{g_r(c_q^2 + h_r^2)^3}\left[\breve{\alpha}_1 \log\left(\frac{(\exp(g_q t) - \tilde{\xi}_1)^2 + \tilde{\xi}_2^2}{(1 - \tilde{\xi}_1)^2 + \tilde{\xi}_2^2}\right) + \breve{\beta}_1 \log\left(\frac{(\exp(g_q t) - \tilde{\xi}_3)^2 + \tilde{\xi}_4^2}{(1 - \tilde{\xi}_3)^2 + \tilde{\xi}_4^2}\right) - \right. \\ & - 2\breve{\alpha}_2 \arctan\left(\frac{\breve{\xi}_2(\exp(g_q t) - 1)}{\breve{\xi}_1^2 + \breve{\xi}_2^2 - \breve{\xi}_1(1 + \exp(g_q t)) + \exp(g_q t)}\right) \\ & \left. - 2\breve{\beta}_2 \arctan\left(\frac{\breve{\xi}_4(\exp(g_q t) - 1)}{\breve{\xi}_3^2 + \breve{\xi}_4^2 - \breve{\xi}_3(1 + \exp(g_q t)) + \exp(g_q t)}\right)\right] \end{aligned} \tag{20}$$

The quantities $\tilde{\xi}_{1,2,3,4}, \tilde{\alpha}_{1,2}, \tilde{\beta}_{1,2}$ can be found from $\xi_{1,2,3,4}, \alpha_{1,2}, \beta_{1,2}$ by substitution of g_r, h_r instead of g_s, h_s in those formulas. Quantities $\breve{\alpha}_{1,2}, \breve{\beta}_{1,2}$ are to be found from $\hat{\alpha}_{1,2}, \hat{\beta}_{1,2}$ in the same way.

To reduce the paper work we may solve numerically the last equation for a number of target protein molecules p upon calculation of four unknowns from (1) in closed form

1.3 Noise and the Hill function analysis

Let us write the non-linear Hill function in a general form: $H(y) = a + (by^2 + ch^2)/(h^2 + y^2)$ to describe both the transcriptional repression or activation by one formal expression.

For example, for the variable q one has $a = 0, b = k_s; c = 0; h = h_s$, for $y = s$ the parameters are $a = g_r; b = g_{max}; c = 0; h = h_g$, while for the repression function the values should be $y = s; a = 0; b == 0; c = k_p; h = h_p$.

The following table and graphs in Figure 1 represent typical behaviour of the function in dependence of time and parameters.

parameters	type	$t \to 0$	$t \to \infty$
$a = 0; c = 0; b \neq 0$	activation	$H \to 0$	$H \to b$
$a \neq 0; b \neq 0; c = 0$	activation	$H \to a$	$H \to a + b$
$a \neq 0; b = 0; c \neq 0$	repression	$H \to a + c$	$H \to a + b$

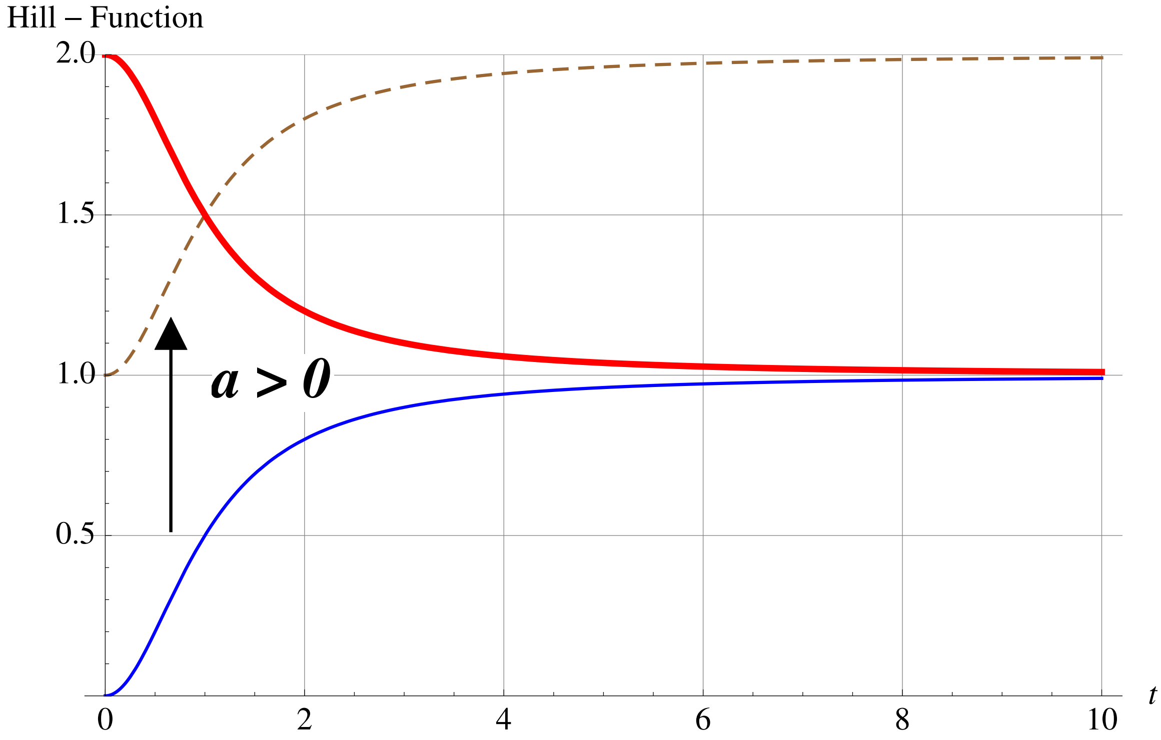

Figure 1: The Hill functions for Activation (Brown dashed graph $a = 1, b = 1, c = 0, h = 1$; and Blue thin graph $a = 0, b = 1, c = 0, h = 1$; graphs) and Repression (Red thick graph $a = 1, b = 0, c = 1, h = 1$;). Time t is in arbitrary units.

Dynamic dependence of the Hill function on parameters may be found via the *Mathematica* package command:
$Manipulate[Plot[a+(bt^2+ch^2)/(h^2+t^2), \{t, 0, 10\}, GridLines- > Automatic, PlotRange- > \{0, 3\}], \{a, 0, 2\}, \{b, 0, 2\}, \{c, 0, 2\}, \{h, 0.1, 2\}]$.

One can see that with a non-zero value of a, e.g., $a = g_r$ a graph is going up without changing its shape, while other parameters are responsible for slope and steady state values of the Hill function. It is worth to note that for those values of an independent time variable

t, for which the Hill sigmoid function is changing rapidly any variation of parameters, e.g., due to noise, will be transferred to a system, but not buffered. For time moments, in which the Hill function value is close to a constant (in Figure 1 t is either close to 0 or $t > 6$) a noise will be buffered.

1.4 The uniqueness proof

In general, the uniqueness of an exact solution to a non-linear ODE may be proven, however it seems to be seldom the case. Fortunately, we can prove it for the system (1) under study.

We consider the system written in form of five coupled equations (1) for an incoherent feed-forward loop (FFL). The difference between coherent and incoherent loops is in form of production function for target mRNA (index r).

The production functions $k_s(q), k_r(q), k_p(s)$ is assumed to be written in the form of the Hill functions: $k(s) = (k_{max}s^c)/(h^c + s^c)$, and describe the production rate controlled by regulators. The values $c = 2$ and $c = -2$ are prescribed for activation and repression, respectively.

For the coherent FFL the production functions will have the following form:

$$k_s(q) = \frac{k_s q^2}{h_s^2 + q^2};\ k_r(q) = \frac{k_r h_r^2}{q^2 + h_r^2};\ k_p(s) = \frac{k_p h_p^2}{s^2 + h_p^2} \tag{21}$$

while for an incoherent FFL the production function for target mRNA will be different:

$$k_r(q) = \frac{k_r q^2}{h_r^2 + q^2}. \tag{22}$$

Let the time variable be from the interval $t \in [0, 1]$, then for each of unknowns $x \in (w, q, s, r, p)$ we prescribe the boundary conditions in the form: $x|_{t=0} = A$, where $A - const$.

Proving the uniqueness of solutions to (1) by contradiction, we assume that each equation in (1) has *two* solutions x_1, x_2, which are different inside the time interval $(0, 1)$ and are *identical* by definition at the initial moment $t = 0$, i.e., at the beginning of a cell cycle. Let us introduce a difference $X = x_1 - x_2$ for each of $x \in (w, q, s, r, p)$, then, obviously $X|_{t=0} = 0$. Writing the first equation from (1) for w_1 and w_2, subtracting one from another we obtain the solution:

$$W = w_1 - w_2 = C\exp(-Dt) = 0 \tag{23}$$

to the equation for W under the initial condition given. For $W = 0$ two second equations, being written for different q_1 and q_2, lead to the same result: $Q = q_1 - q_2 = 0 \to q_1 = q_2$. Similarly, for the third equations we have for $S = s_1 - s_2$:

$$S_t = \frac{k_s h_s^2}{(h_s^2 + q_1^2)(h_s^2 + q_2^2)} Q(q_1 + q_2) - g_s S. \tag{24}$$

which for $Q = 0$ yields now $S = 0$ similar to (23). The fourth equation for $R = r_1 - r_2$ is reduced to:

$$R_t = k_r h_r^2 \frac{q_2^2 - q_1^2}{(h_r^2 + q_1^2)(h_r^2 + q_2^2)} - g_r R \tag{25}$$

and for $Q = 0$ we obtain $R = 0$. Eventually, for $P = p_1 - p_2$ we have:

$$P_t = k_p h_p^2 \frac{s_2^2 - s_1^2}{(h_p^2 + s_1^2)(h_p^2 + s_2^2)} - g_p P = -g_p P \tag{26}$$

that means $P = 0$. Therefore the solution to the coupled ODE (1) is *unique* for every set of initial conditions given.

The similar proof is valid for both the linear circuit (4 equations) and the open circuit (7 equations) (see Osella et al., (2011)) because both systems do not contain any nonlinearity except the Hill functions already written in (1).

The model with non-linear degradation of target mRNA contains the same equations for w, q, s, p as in (1), while the equation for r has different nonlinearity:

$$\frac{dr}{dt} = k_r(q) - g_r(s)r = \frac{k_r q^2}{h_r^2 + q^2} - \left(g_r + \frac{g_{max} s^2}{h_r^2 + s^2} \right) r. \tag{27}$$

Calculating the difference $R = r_1 - r_2$ between two tentative solutions, we obtain an equation:

$$R_t = \frac{k_r h_r^2 Q(q_1 + q_2)}{(h_r^2 + q_1^2)(h_r^2 + q_2^2)} - g_r R - \frac{g_{max}}{(h_r^2 + s_1^2)(h_r^2 + s_2^2)} \left(h_r^2(s_1^2 r_1 - s_2^2 r_2) + s_1^2 s_2^2 (r_1 - r_2) \right) \tag{28}$$

which for $Q = S = 0 \Rightarrow s_1 = s_2$ yields the equation:

$$R_t = -g_r R - R(s_1^4 + h_r^2 s_1^2) \frac{g_{max}}{(h_r^2 + s_1^2)^2} = -g_r\ R - R\ s_1^2 \frac{g_{max}}{(h_r^2 + s_1^2)}, \tag{29}$$

having a solution in the form:

$$R(t) = C \exp\left(-g_r t - g_{max} \int_0^t \frac{s_1^2 dt}{h_r^2 + s^2} \right). \tag{30}$$

¿From the initial condition $R|_{t=0} = 0$ we have $C = 0$, and $R = r_1 - r_2 = 0 \Rightarrow r_1 = r_2$, therefore this model equation has the unique solution, too.

The last equation for $p(t)$ of the model has the form:

$$\frac{dp}{dt} = k_p(s)r - g_p p \tag{31}$$

and similar approach together with $R = 0$ leads to the conclusion $P = 0$, i.e., the solution to (31) is unique.

Hence, the solution to the coupled model equations *with non-linear Hill's degradation* of target mRNA is unique.
