# Supplementary material for: Dynamics of miRNA driven feed-forward loop depends upon miRNA action mechanisms
Source: BMC Genomics. 2014 Dec 19;15(Suppl 12):S9. doi: 10.1186/1471-2164-15-S12-S9 (PMC4303955; doi:10.1186/1471-2164-15-S12-S9)
Supplement: Additional file 2 — contains additional figures useful for better understanding the results presented in the main text. [file 1471-2164-15-S12-S9-S2.pdf]

1 Additional figures

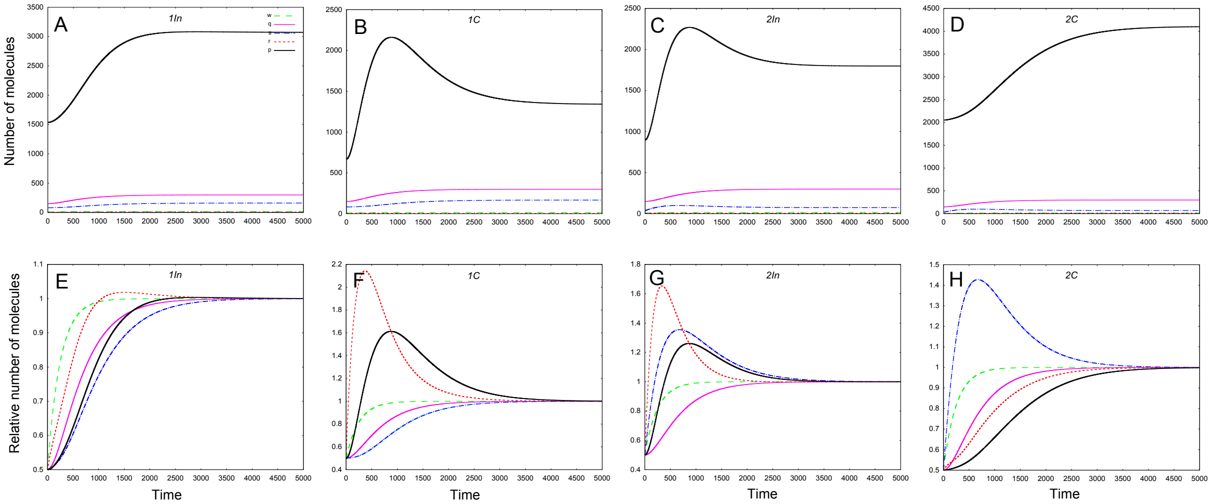

Figure 1: The solutions to the Dual degradation model in various FFLs. 1In - type 1 incoherent FFL, 1C- type 1 coherent FFL, 2In - type 2 incoherent FFL, 2C - type 2 coherent FFL; w, q, s, r and p denote graphs of solutions for TF mRNA, TF, miRNA, target mRNA and target protein, resp. **A-D :** temporal dynamics of absolute number of each molecule species is presented. **E-H:** molecules numbers for each species are normalized on steady state values to better visualize the behavior of RNA species. **A and E:** the solution to the Dual degradation model in type 1 incoherent FFL. **B and F:** the solution to the Dual degradation model in type 1 coherent FFL. **C and G:** the solution to the Dual degradation model in type 2 incoherent FFL. **D and H:** the solution to the Dual degradation model in type 2 coherent FFL.

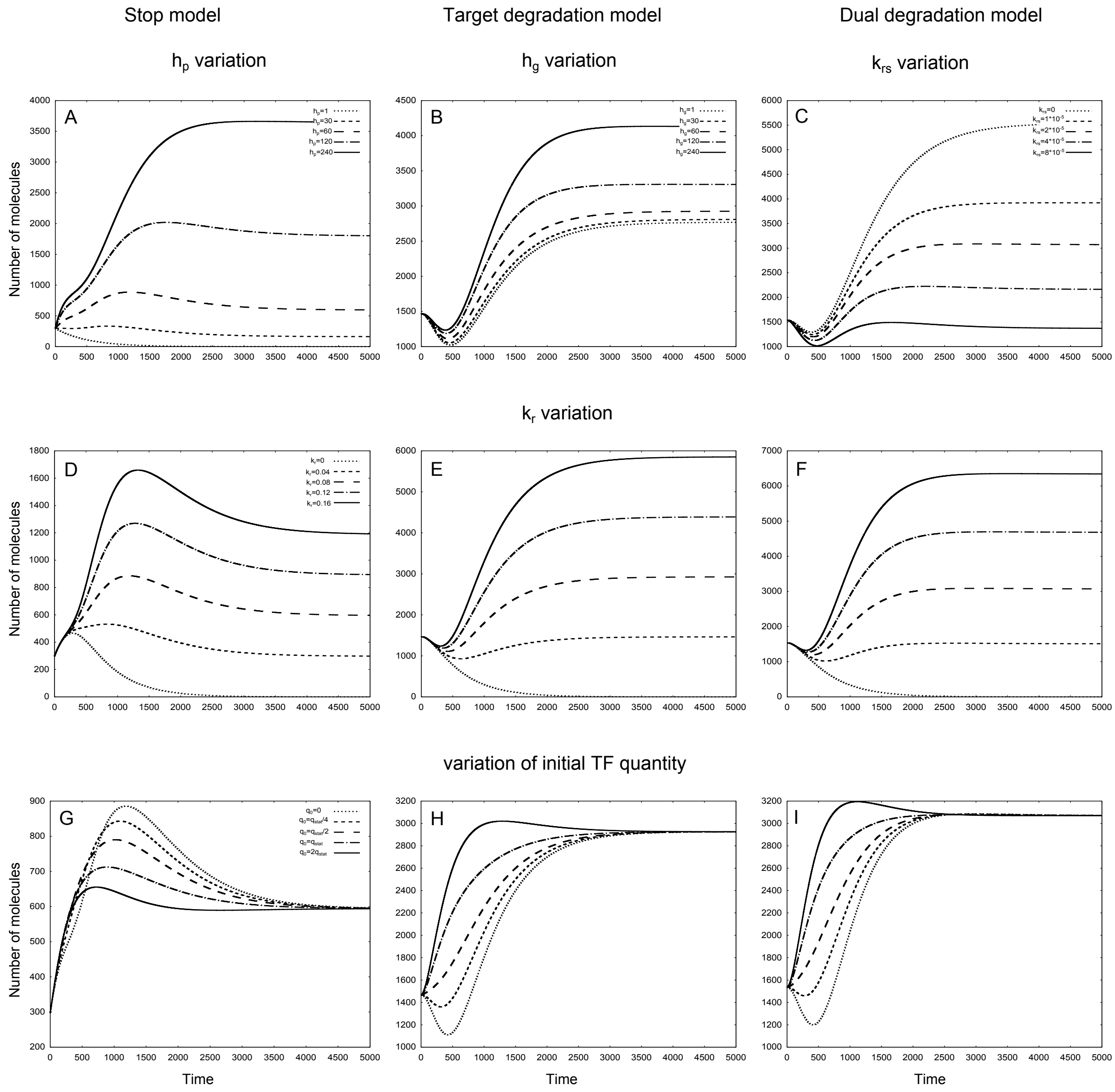

Figure 2: The variation of target protein quantities in different models of $1In$ loop in response to variation of h_p, h_g, k_rs, k_r and initial TF quantities. The parameters h_p, h_g, k_rs define the action of miRNA on target mRNA, k_r is a maximal rate of transcription for target mRNA. Left column - Stop model, central column - Target degradation model, right column - Dual degradation model. **A :** In the Stop model the quantity of target protein increases as the value of h_p parameter increases from 0 to 240 molecules. **B:** In the Target degradation model the quantity of target protein increases as the value of h_g parameter increases from 0 to 240 molecules. **C:** The gradual increase of the k_{rs} coefficient from 0 to 8×10^{-5} $mol^{-1}sec^{-1}$ results in the fall of the target protein quantity in the Dual degradation model. **D - F:** In all models the gradual increase of k_r value within the (0, 0.16) interval results in the increase of the target protein production. **G - I:** In all models the variation of initial number of TF molecules does not change the target protein quantities at steady state. Initial quantities of TF were changed as described in section "Methods"

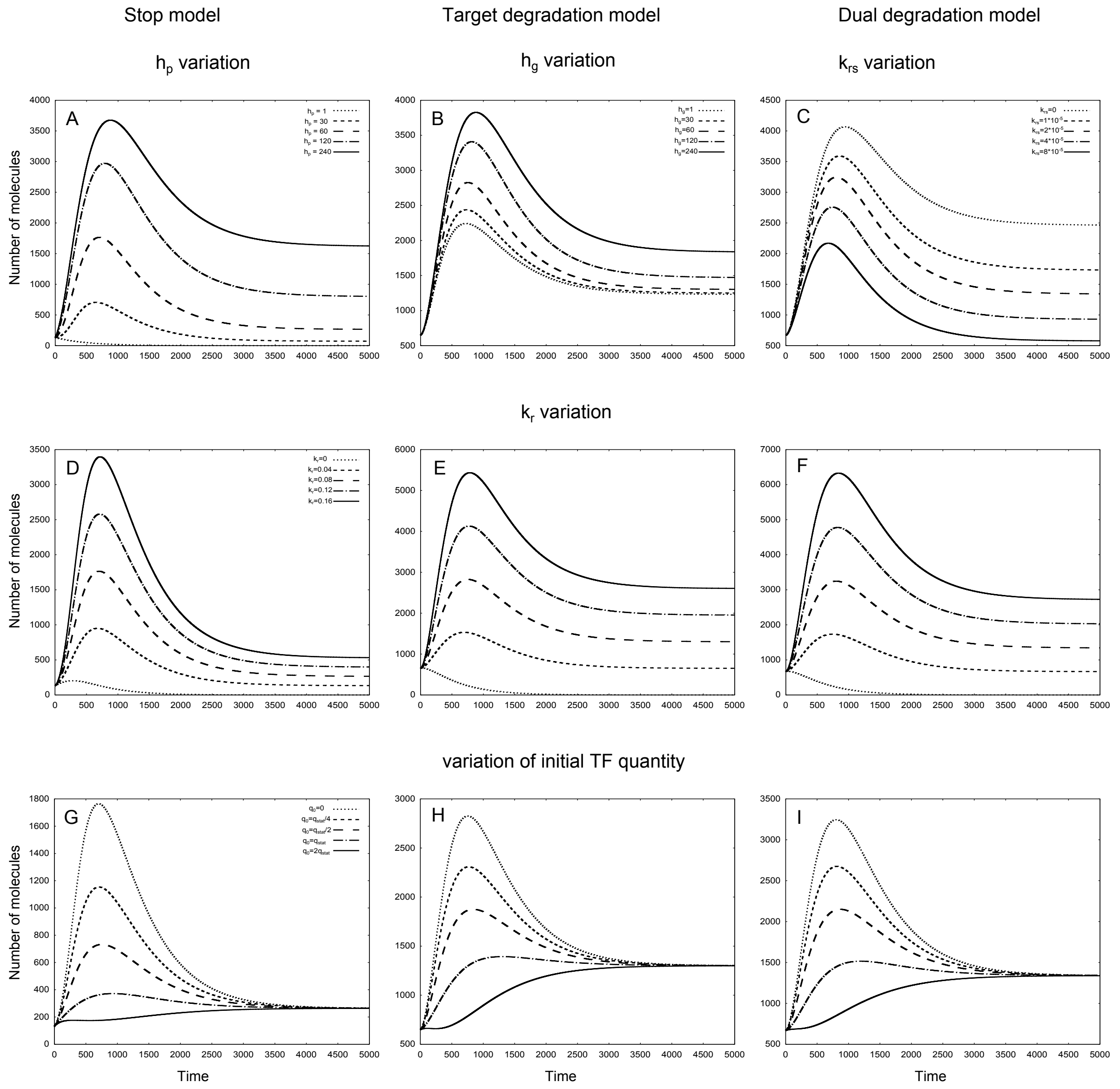

Figure 3: (The variation of target protein quantities in different models of $1C$ loop in response to variation of h_p, h_g, k_rs, k_r and initial TF quantities. The parameter values are the same as for Fig A2. Left column - Stop model, central column - Target degradation model, right column - Dual degradation model. **A:** The increase of h_p coefficient results in the increase of the target protein quantity. **B:** Only the large values of h_g have a noticeable effect on the target protein production. **C:** In the Dual degradation model the increase of k_{rs} results in the fall down of the target protein quantity. **D - F:** The increase of k_r coefficient results in increase of the target protein quantities in all models. **G - I:** The increase of the initial number of TF molecules results in the transformation of the bell-shaped target protein profile into uprising curve, tending to a constant value in all models.

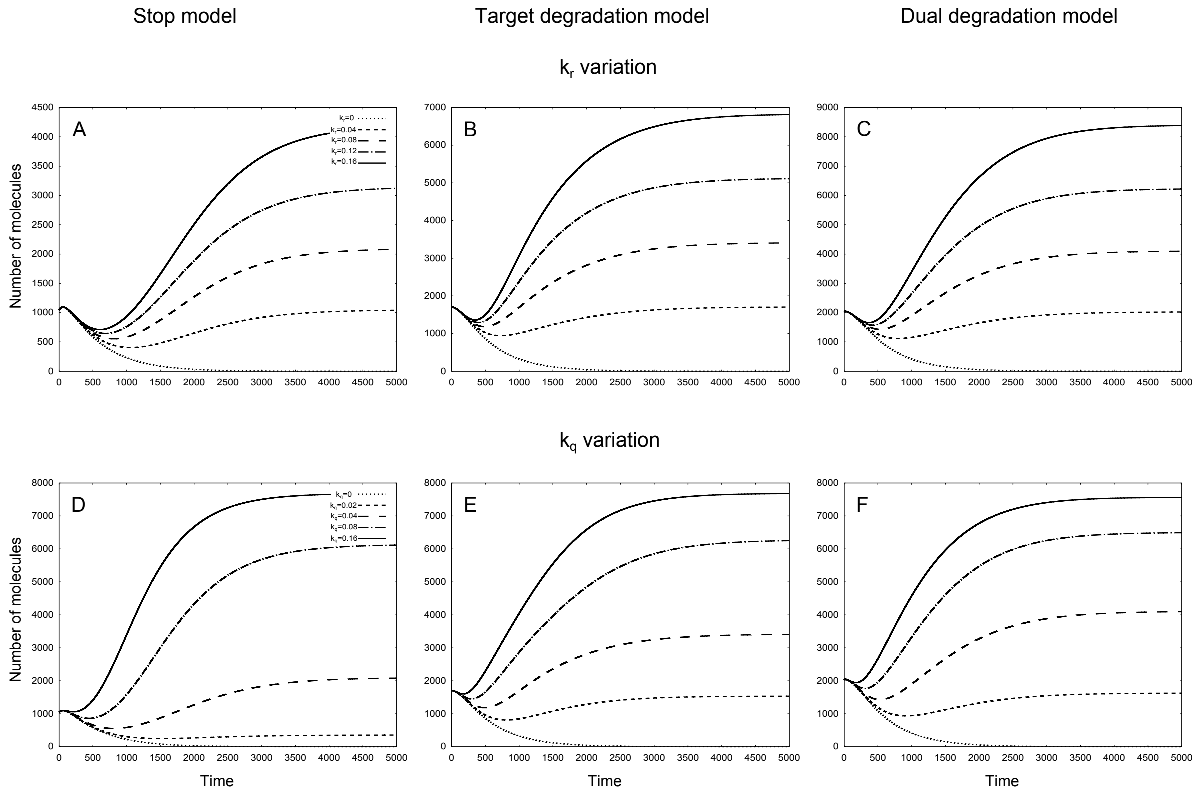

Figure 4: The variation of target protein quantities in different models of $2C$ loop in response to variation of k_q and k_r coefficients. The k_q and k_r coefficients define TF translation rate and maximal rate of target mRNA transcription correspondingly. The parameter values are the same as of Fig. A2 and Fig.4 in main text. Left column - Stop model, central column - Target degradation model, right column - Dual degradation model. **A -C:** In all models the increase of k_r results in the increase of the target protein quantity. **D - F:** The patterns of dependence of target protein quantities on k_q variation.

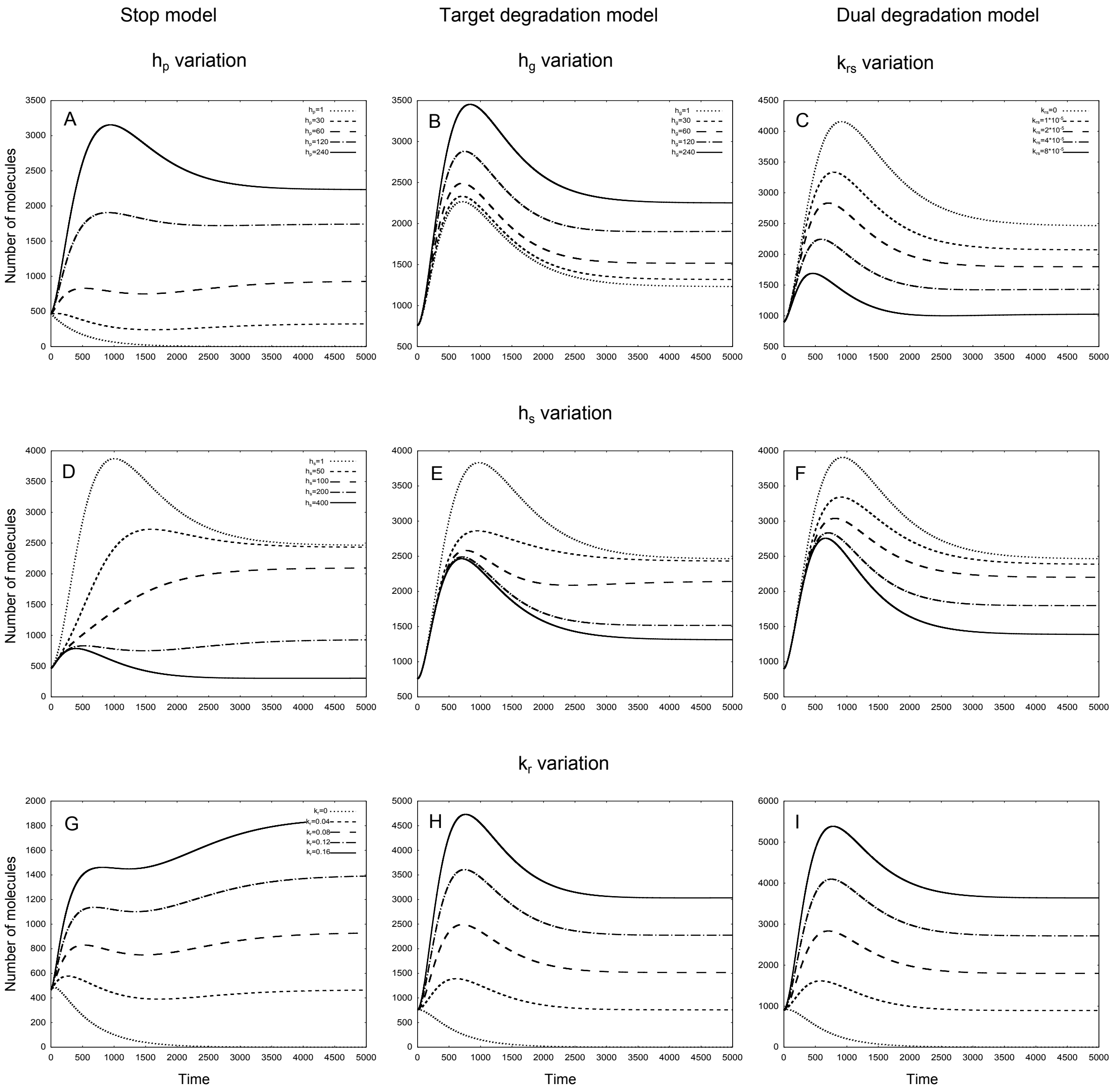

Figure 5: The variation of target protein quantities in different models of $2In$ loop in response to variation of h_p, h_g, k_rs, h_s and k_r coefficients. The h_p, h_g, k_rs coefficients define the action of miRNA on target mRNA, h_s is the amount of TFs, at which the transcription rate of miRNA gene is half of its maximum value, k_r coefficient is the maximal rate of transcription for target mRNA gene. The parameter values are the same as of Fig A2 and Fig.4 in main text. Left column - Stop model, central column - Target degradation model, right column - Dual degradation model. **A - B:** The increase of h_p coefficient in the Stop model and h_g coefficient in the Target degradation model results in the target protein quantities growth. **C:** Growth of k_{rs} parameter results in fall of the target protein quantity in the Dual degradation model. **D - F:** In all models the increase of the h_s coefficient results in decrease of the target protein quantities. **G - I**: In all models the increase of the k_r coefficient values result in the increase of the target protein quantities.

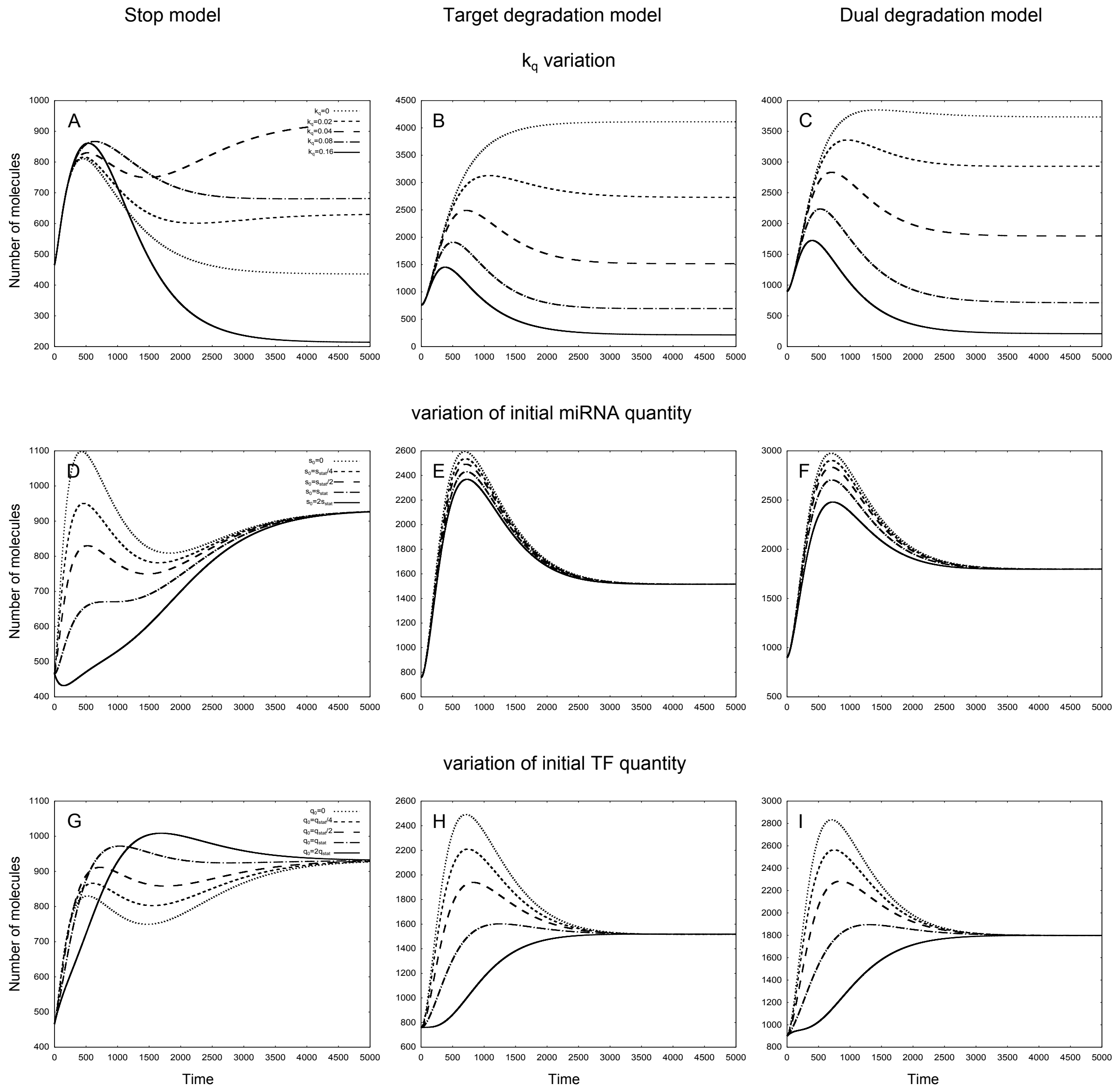

Figure 6: The variation of target protein quantities in different models of $2In$ loop in response to variation of k_q coefficient and initial miRNA and TF quantities. The parameter values are the same as of Fig. A2 and Fig.4 in main text. Left column - Stop model, central column - Target degradation model, right column - Dual degradation model. **A:** In the Stop model the largest number of target protein molecules at steady state is observed for intermediate values of k_q. **B - C :** In the Target degradation and the Dual degradation models the increase of the TF translation rate results in the fall of target protein quantities. **D -F:** The patterns of dependence of target protein profiles on variation of initial miRNA quantities in the models. **G - I:** The patterns of dependence of target protein profiles on variation of initial TF quantities in the models.
